# The Proximity of Ribosomal Protein Genes to *oriC* Enhances *Vibrio cholerae* Fitness in the Absence of Multifork Replication

**DOI:** 10.1128/mBio.00097-17

**Published:** 2017-02-28

**Authors:** Alfonso Soler-Bistué, Michaël Timmermans, Didier Mazel

**Affiliations:** a Département Génomes et Génétique, Institut Pasteur, Unité Plasticité du Génome Bactérien, Paris, France; b Centre National de la Recherche Scientifique UMR3525, Paris, France; The Sanger Institute

## Abstract

Recent works suggest that bacterial gene order links chromosome structure to cell homeostasis. Comparative genomics showed that, in fast-growing bacteria, ribosomal protein genes (RP) locate near the replication origin (*oriC*). We recently showed that *Vibrio cholerae* employs this positional bias as a growth optimization strategy: under fast-growth conditions, multifork replication increases RP dosage and expression. However, RP location may provide advantages in a dosage-independent manner: for example, the physical proximity of the many ribosomal components, in the context of a crowded cytoplasm, may favor ribosome biogenesis. To uncover putative dosage-independent effects, we studied isogenic *V. cholerae* derivatives in which the major RP locus, *S10-spc-α* (S10), was relocated to alternative genomic positions. When bacteria grew fast, bacterial fitness was reduced according to the S10 relative distance to *oriC*. The growth of wild-type *V. cholerae* could not be improved by additional copies of the locus, suggesting a physiologically optimized genomic location. Slow growth is expected to uncouple RP position from dosage, since multifork replication does not occur. Under these conditions, we detected a fitness impairment when S10 was far from *oriC*. Deep sequencing followed by marker frequency analysis in the absence of multifork replication revealed an up to 30% S10 dosage reduction associated with its relocation that closely correlated with fitness alterations. Hence, the impact of S10 location goes beyond a growth optimization strategy during feast periods. RP location may be important during the whole life cycle of this pathogen.

## IMPORTANCE

The role of gene order within the bacterial chromosome is poorly understood. In fast growers, the location of genes linked with the expression of genetic information (i.e., transcription and translation) is biased toward *oriC*. It was proposed that the location of these genes helps to maximize their expression by recruiting multifork replication during fast growth. Our results show that such genomic positioning impacts cell fitness beyond fast-growth conditions, probably across the whole life cycle of fast growers. Thus, the genomic position of key highly expressed genes, such as RP, was finely tuned during the evolution of fast-growing bacteria and may also be important in slow growers. In the near future, many more genes whose genomic position impacts bacterial phenotype will be described. These studies will contribute to discover the rules of genome organization and application of them for the design of synthetic chromosomes and the creation of artificial life forms.

## INTRODUCTION

**FIG 1:**
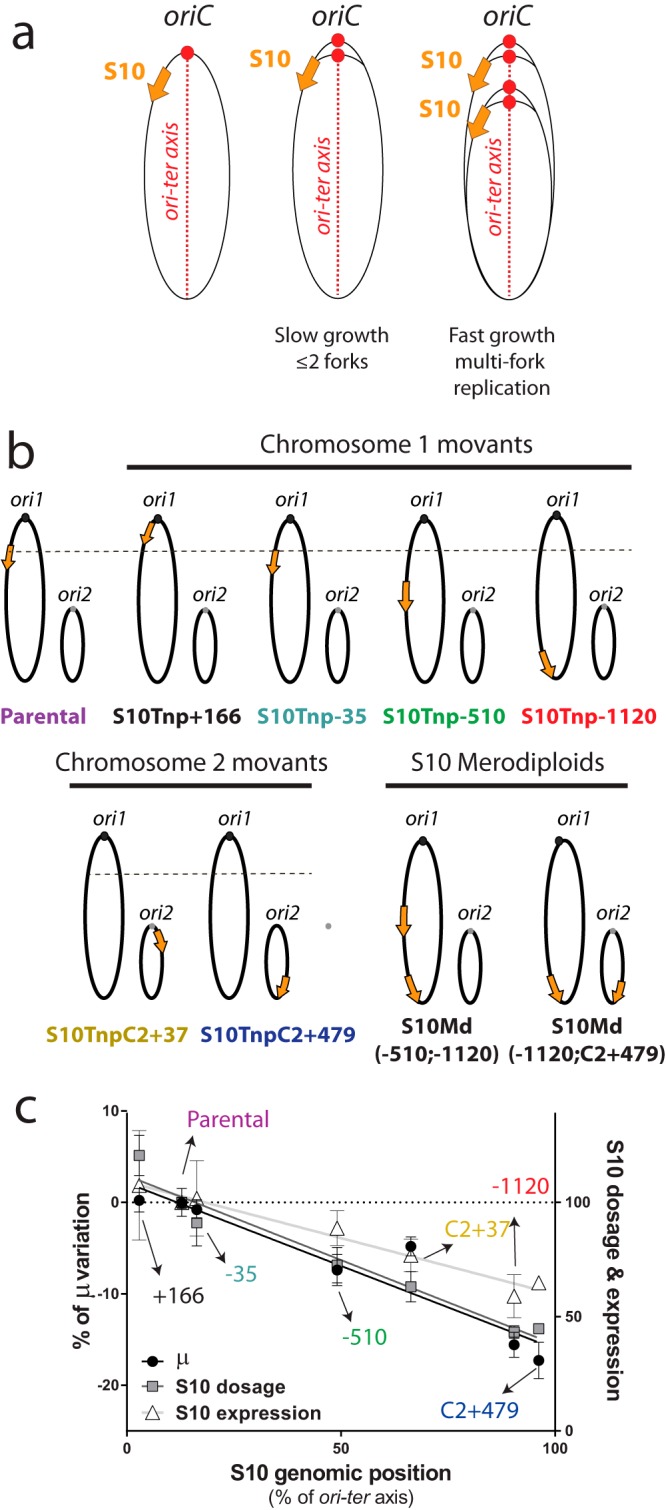
Links between genome organization, S10 location, and cell physiology. (a) The existence of a single *oriC* (red circle) organizes the bacterial genome across an *ori*-*ter* axis (left). In exponential phase, fast-growing bacteria overlap replication rounds, increasing the dosage of *oriC*-neighboring regions (right). This impacts S10 genome-wide copy number (orange arrow). (b) Genome organization of the strains used in this study showing the parental, movant, and merodiploid strains. *ori1* and *ori2* are depicted as dark and light gray dots, respectively. The orange arrow represents S10 according to its genomic position and ploidy. The dashed line represents the S10 location in the parental strain. Chromosomes are drawn according to their replication timing. (c) The maximum growth rate (µ, black dots) and the relative S10 dosage (gray squares) and expression (white triangles) with respect to the parental strain were plotted as a function of S10 relocation along the *ori*-*ter* axis in the *V. cholerae* genome. S10 location, dosage, expression, and the µ closely correlate.

An increasing body of evidence shows that bacterial gene order contributes to harmonizing genome organization with cellular physiology (1–16). Bacteria possess a single origin of replication (*oriC*) from which replication starts bidirectionally until the replication forks meet at the terminus region of the chromosome (*ter*). This organizes the genome into two equally sized replichores along the *ori-ter* axis (Fig. 1a, left). Genes coding for the expression of genetic information (i.e., transcription and translation) tend to be located close to *oriC* only in fast-growing bacteria (4, 13). During exponential phase under optimal conditions, when bacteria constantly have their highest replication rate, fast growers display a generation time that is shorter than the time needed to replicate their genetic material. To cope with this, bacteria overlap replication rounds by firing *oriC* on partially replicated chromosomes, a process called multifork replication (Fig. 1a, right). For example, *Pectobacterium carotovorum* might potentially have up to 30 replication forks within a single cell (4). As a consequence, during the exponential phase under fast-growth conditions, genes close to *oriC* benefit from a higher dosage. During this stage, ribosome number and transcriptional activity attain their maximum (17). It has been proposed that the location bias observed in ribosomal and RNA polymerase (RNAP) genes has been selected during evolution to benefit from multifork replication, increasing their copy number when most needed (13, 18). Indeed, when replication-associated gene dosage effects (*R*) were estimated among hundreds of bacterial species, a tight inverse correlation between *R* and generation time emerged (4). This means that the fastest-growing bacteria are those capable of achieving higher levels of multifork replication. Hence, there is a strong link between ribosomal and RNAP genomic location, *R*, and growth rate.

We recently tackled this issue in *Vibrio cholerae*, a Gram-negative human pathogen displaying one of the highest growth rates, using a positional genetics approach (19). *V. cholerae* is a model for studying bacteria with multiple chromosomes (20) since it possesses a main chromosome (Chr1) of 2.96 Mbp and a secondary chromosome (Chr2) of 1.07 Mbp. Genome replication starts at the origin of Chr1 (*ori1*), and the origin of Chr2 (*ori2*) fires when two-thirds of the larger replicon has been duplicated, finishing their replications synchronously (21, 22). The *V. cholerae* life cycle alternates between an amplification period during host infection and a persistence phase in the environment (23). Fast growth is associated with the amplification period while slow growth resembles the environmental conditions of the persistence phase. We manipulated the genome of this bacterium to move an essential (24), widely conserved (25) 13.2-kbp locus harboring half of the ribosomal protein genes (RP), *S10-spc-α* (S10), to several genomic locations. Thus, we created a series of isogenic movant (6) strains (i.e., isogenic strains where the genomic position of a specific locus changes) to study the link between the genomic location of S10 and cell physiology (Fig. 1b). Under fast-growth conditions, we found that the maximum growth rate (µ) was reduced as a function of the distance between S10 and *ori1* (Fig. 1c). Changes in μ tightly correlated with S10 dosage and expression. Importantly, S10 merodiploid strains having two copies far away from *oriC* displayed a normal μ, demonstrating that, during fast growth, the dosage of RP rather than its genomic location *per se* was essential for cell physiology. In line with this, we did not observe μ alterations under slow-growth conditions (19). These experiments cannot rule out that the biased position of RP and RNAP could also be the result of other evolutionary forces not relying on *oriC*-linked dosage. For instance, it has recently been shown that, among other loci, S10 is important for structuring the chromosome by limiting domain boundaries in *Caulobacter crescentus* (26, 27). Also, S10 genomic position enables its physical proximity to many other ribosomal genes, 9 rRNA operons, and more than 50 different RP (21). This might be crucial for ribosome assembly since, after transcription, bacterial mRNAs remain near their transcription sites (7) in the context of a cell cytoplasm possessing a high concentration of macromolecules that constrain molecular interactions and hamper fast diffusion (28–32).

In this paper, we aimed at uncovering such dosage-independent effects. First, we addressed putative fitness effects of S10 genomic position in *Vibrio cholerae* by analyzing movant strains by pairwise competition, a more sensitive method than growth curves (33). Second, we studied movant strains under slow-growth conditions to permit the completion of chromosomal replication before cell division. Thus, we expected to uncouple S10 subcellular position from dose changes, allowing dosage-independent effects to be observed. Under these conditions, relocation of S10 far from *oriC* showed an impaired competition capacity. Despite the absence of multifork replication, we observed significant S10 dosage differences that correlated with fitness alterations. Contrary to what we expected, we found that slight S10 genome-wide copy number alterations impacted *V. cholerae* fitness, indicating the high importance of its genomic positioning during the whole life cycle of this pathogen.

## RESULTS

### S10 relocation impacts *V. cholerae* fitness under fast-growth conditions.

**FIG 2:**
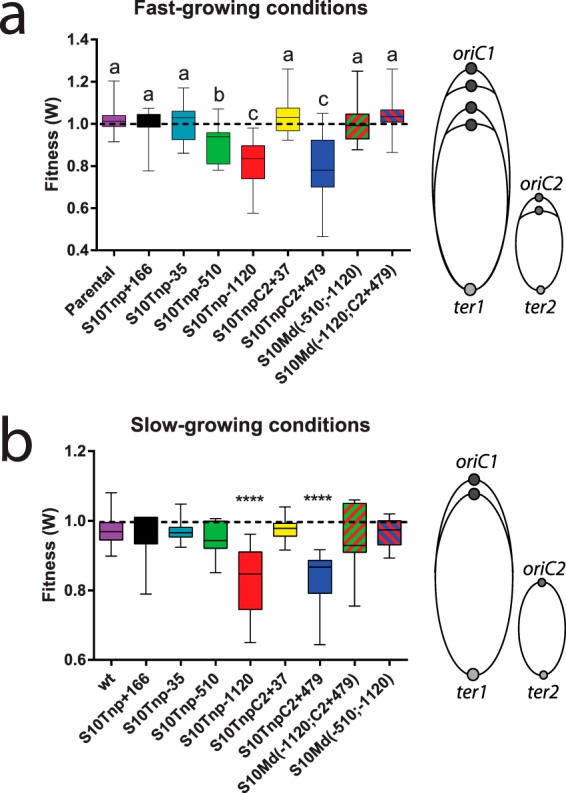
Movant strain fitness in pairwise competition experiments. S10 relocation effect on fitness was assessed by averaging the obtained *W* values against Parental−1120::gfpmut3* of the parental, movant, and merodiploid strains. Results are shown in standard box-and-whisker plots showing the medians, minima, and maxima of *W* values. Statistical significance was analyzed using nonparametric tests and the Dunn test for multiple comparisons of the means obtained for each strain. (a) Competition experiments done under fast-growth conditions when overlapping replication rounds occur as depicted in the right panel. Letters denote groups showing statistically significant differences (*P* of at least <0.01). (b) Competition experiments performed under slow-growth conditions in the absence of multifork replication as depicted in the right panel. ****, *P* < 0.0001. The rest display no statistically significant differences with respect to the parental strain.

In prior work (19), μ was assessed over a set of movant strains in which the S10 locus was relocated to different genomic positions (Fig. 1b). We showed that multifork replication led to S10 dosage differences that were crucial for physiology under fast-growth conditions (Fig. 1c). To unravel putative dosage-independent effects for which µ could be not sensitive enough (33), we performed pairwise competition assays. For this, we cocultured equal amounts of the parental green fluorescent protein (GFP)-tagged *V. cholerae* (see Table S1 in the supplemental material) and each one of the unmarked strains. Then, we monitored deviations from a 1:1 ratio to measure the absolute fitness (*W*). Under fast-growth conditions, we detected a statistically significant fitness decrease in strains where S10 is relocated far from *ori1* (Fig. 2a; Table 1). We observed a distance-dependent fitness reduction since the strains where S10 is located furthest from *ori1*, S10Tnp−1120 and S10TnpC2+479 movants, showed a stronger effect (*W* = 0.82 ± 0.1 and *W* = 0.8 ± 0.13, respectively) than the strain in which this locus is located in the middle of the replichore, S10Tnp−510 (*W* = 0.9 ± 0.08). Meanwhile, S10Tnp−35, the movant in which S10 was slightly relocated, displayed a similar *W* value as the parental strain (*W* = 1.0 ± 0.07), showing that S10 relocation *per se* did not impact cell fitness. S10Tnp+166 presented no significant fitness alterations (*W* = 1.0 ± 0.07), suggesting that (i) a small S10 dosage increase is not toxic for the cell and (ii) S10 dosage is not limiting cell growth. To uncouple S10 dosage effects from putative effects due to changes in the subcellular location, we studied merodiploid strains carrying two S10 copies far away from *ori1*, S10Md(−510;−1120) and S10Md(−1120;C2+479) (Fig. 1b). These two mutants displayed no fitness impairment compared to the parental strain (*W* = 1.0 ± 0.09 and *W* = 1.03 ± 0.1, respectively). Since S10 copy number recovery abolished the fitness handicap independently of its genomic location, S10 dosage must be the main mechanism behind the observed effect. Overall, these results are in line with previous μ measurements (Table 1). However, S10TnpC2+37 and S10Md(−1120;C2+479), two strains presenting a reduced µ, did not display a fitness reduction compared to the parental strain (*W* = 1.04 ± 0.08 and *W* = 1.03 ± 0.1, respectively).

10.1128/mBio.00097-17.4 TABLE S1  Full list of plasmids and bacterial strains used. Download TABLE S1, PDF file, 0.2 MB. Copyright © 2017 Soler-Bistue et al. 2017 Soler-Bistue et al. This content is distributed under the terms of the Creative Commons Attribution 4.0 International license.

**TABLE 1:** Relative fitness and growth rate variation of movant strains under fast-growth conditions^b^

Strain	*W*_rel_	% of μ variation^a^
S10Tnp+166	0.989 ± 0.103	0.22 ± 1.89
S10Tnp−35	0.993 ± 0.045	−0.79 ± 1.85
S10Tnp−510	0.895 ± 0.066	−7.39 ± 2.67
S10Tnp−1120	0.791 ± 0.126	−15.58 ± 3.14
S10TnpC2+37	0.998 ± 0.053	−4.8 ± 1.8
S10TnpC2+479	0.787 ± 0.158	−17.29 ± 3.44
S10Md(−510;−1120)	0.981 ± 0.055	−3.07 ± 3.59
S10Md(−1120;C2+479)	1.012 ± 0.071	−4.58 ± 3.48

a Data from the work of Soler-Bistué et al. (19).

b The fitness of *V. cholerae* derivatives was measured by pairwise competition. Results are shown as *W*_rel_ ± 95% confidence interval with respect to the parental strain. The percentage of μ variation with respect to the parental strain is included for comparative purposes.

### Effects of an artificially increased S10 ploidy.

**FIG 3:**
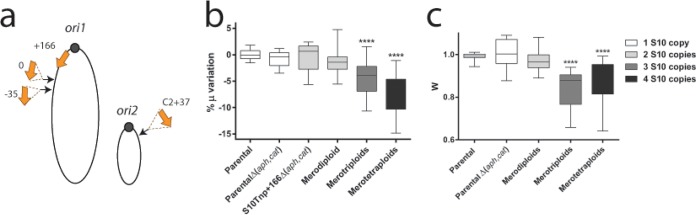
Impact of additional S10 copies in *V. cholerae*. (a) Several S10 copies (orange arrows) were inserted within the S10Tnp+166Δ(*aph*,*cat*) genome. Their insertion sites, drawn as black arrows and the coordinates (in kilobase pairs from the original location), are shown within *V. cholerae* chromosomes. (b) The S10 ploidy effect on growth rate was quantified by averaging obtained μ in at least 3 independent experiments, with 3 or more biological replicates, for each mutant strain and normalizing it to the μ of the parental strain. Results are expressed as percentage of the variation (% μ) with respect to parental strains. (c) Competition experiments done under fast-growth conditions. For panels b and c, data are shown using box-and-whisker plots. Statistical significance was analyzed by a nonparametric test, and the Dunn test was done to compare the mean values obtained for each strain. ****, *P* < 0.0001.

Curiously, S10Tnp+166, the strain having the highest S10 dosage, did not show phenotypic alterations (Table 1). Hence, we inquired about the possible effects of further increasing S10 copy number. We inserted S10 at positions (i) showing no fitness alterations and (ii) as close as possible to *ori1*, allowing a maximal genome-wide copy number (Fig. 3a). S10 ploidy was verified by Southern blotting (Fig. S1). We named S10 meropolyploids (M followed by S10 copy number) according to the position in which the loci were inserted according to previously established nomenclature for coordinates (19). For instance, the obtained merotetraploid is S10M4(+166;0;−35;C2+37). These strains displayed no obvious defects since they presented a regular colony morphology, a normal cell shape, and no viability loss (data not shown). To further characterize the meropolyploids, growth curves were performed to determine the μ of these mutants (Fig. 3b). First, S10 merodiploid strains showed a similar growth as the parental strain. No statistically significant differences were observed either within merodiploids [S10M2(+166;0), S10M2(+166;−35), and S10M2(+166;C2+37)] or within merotriploids [S10M3(+166;0;−35) and S10M3(+166;0;C2+37)], indicating that ploidy rather than position is responsible for growth reduction (Fig. S2). Meanwhile, the addition of a third and fourth locus copy impacted cell growth, since merotriploid and merotetraploid strains showed μ reductions of 4.74% ± 2.16% and 6.99% ± 3.56% with respect to the parental strain. Pairwise competition assays (Fig. 3c) showed similar results. Merodiploids showed no fitness defect (*W* = 0.973 ± 0.05). Meanwhile, merotriploids and merotetraploid strains display a lower fitness than parental strains (*W* = 0.85 ± 0.07 and *W* = 0.85 ± 0.1, respectively). We conclude that μ cannot be further improved by increasing S10 dosage, showing that genome-wide copy number of these genes is not limiting for growth. The cell can tolerate putative detrimental effects of an extra copy, but increasing S10 ploidy beyond two, such as in merotriploid and merotetraploid strains, impairs cell physiology. Overall, these results suggest that S10 is already at its optimal position to ensure the best growth of *V. cholerae*.

10.1128/mBio.00097-17.2 FIG S1  Southern blot analysis of EcoRV-digested gDNA of the strains indicated in the upper panel. Probes were targeted to the *rpsJ* gene (red, DY682) or to the Zeo^r^ marker linked to the parental *rpsJ* gene from the PGB-B393 strain (green, DY782). Genotype changes are evidenced by size change of S10 upon movement (parental versus S10Tnp+166). Then, the addition of the second and third copies from S10Tnp−35Δ*aph* and S10TnpC2+37Δ*cat* (black arrows). The fourth S10 copy comes from PGB-B393. The restriction fragment has a similar size as the +166 allele. However, it can be distinguished by an increased S10 probe signal (red arrow) and the green band. S10 ploidy of each strain is shown in the lower panel. Download FIG S1, PDF file, 0.1 MB. Copyright © 2017 Soler-Bistue et al. 2017 Soler-Bistue et al. This content is distributed under the terms of the Creative Commons Attribution 4.0 International license.

10.1128/mBio.00097-17.3 FIG S2  Analysis of growth rate of meropolyploids. The S10 ploidy effect on GR was quantified by averaging obtained μ in at least 2 independent experiments, with 4 or more biological replicates, for each mutant strain and normalizing it to the μ of the parental strain. Results are expressed as percentage of the variation (μ %) with 95% confidence interval with respect to parental strains. Statistical significance was analyzed by one-way analysis of variance (two-tailed test). Then, the Dunn test was used for multiple comparisons, taking the parental strains as control. Statistically significant differences are indicated (***, *P* < 0.001; ****, *P* < 0.0001). Download FIG S2, PDF file, 0.1 MB. Copyright © 2017 Soler-Bistue et al. 2017 Soler-Bistue et al. This content is distributed under the terms of the Creative Commons Attribution 4.0 International license.

### S10 position impacts *V. cholerae* fitness under slow-growth conditions.

In the text above, dosage-independent effects were not detected in rich medium (Fig. 2a). To uncover them, we performed pairwise competition under slow-growth conditions of the whole strain set (Fig. 1b) against the GFP-tagged parental strain. Under these conditions, we expected to avoid multifork replication, uncoupling S10 subcellular location from its dosage (Fig. 1a, center). The results depicted in Fig. 2b and Table 2 showed that no fitness cost was associated with the relocation of S10 closer to *oriC* (S10Tnp+166, *W* = 0.998 ± 0.088), a few kilobase pairs away from its original location (S10Tnp−35, *W* = 0.998 ± 0.04), to the middle of the left replichore of Chr1 (S10Tnp−510, *W* = 0.987 ± 0.075), or to Chr2, close to its replication origin (S10TnpC2+37, *W* = 1.01 ± 0.04). This indicates that the precise location of the major ribosomal protein locus and the possible associated structural alterations of nucleoid structure are irrelevant for *V. cholerae* fitness. Interestingly, S10Tnp−1120 and S10TnpC2+479, the movants in which S10 is located the furthest away from *ori1* and the rest of their functional partners, showed a highly significant fitness reduction (*W* = 0.858 ± 0.109 and *W* = 0.846 ± 0.094, respectively). This suggested the existence of dosage-independent effects. To confirm this, we analyzed S10Md(−510;−1120) and S10Md(−1120;C2+479), strains bearing two copies of S10 far from *ori1*. If the S10 subcellular location were necessary for an optimal fitness, these strains should also present a reduction in *W*. Surprisingly, these derivatives rescued fitness defects (*W* = 0.97 ± 0.079 and *W* = 0.978 ± 0.082, respectively), indicating that dosage-independent effects of S10 genomic position are very mild or nonexistent. Therefore, although in minimal medium dosage differences should be negligible, they constitute the most plausible explanation for the observed phenotypes.

**TABLE 2:** Fitness of movant strains under slow-growth conditions^a^

Strain	*W*
S10Tnp+166	0.998 ± 0.088
S10Tnp−35	0.998 ± 0.04
S10Tnp−510	0.987 ± 0.075
S10Tnp−1120	0.858 ± 0.109
S10TnpC2+37	1.01 ± 0.04
S10TnpC2+479	0.846 ± 0.094
S10Md(−510;−1120)	0.97 ± 0.079
S10Md(−1120;C2+479)	0.978 ± 0.082

a Fitness of *V. cholerae* derivatives measured by pairwise competition against the parental strain carrying gfpmut3*. Results are shown as *W* ± 95% confidence interval.

### S10 relocation causes dosage differences in the absence of overlapping replication rounds correlating with fitness loss.

**FIG 4:**
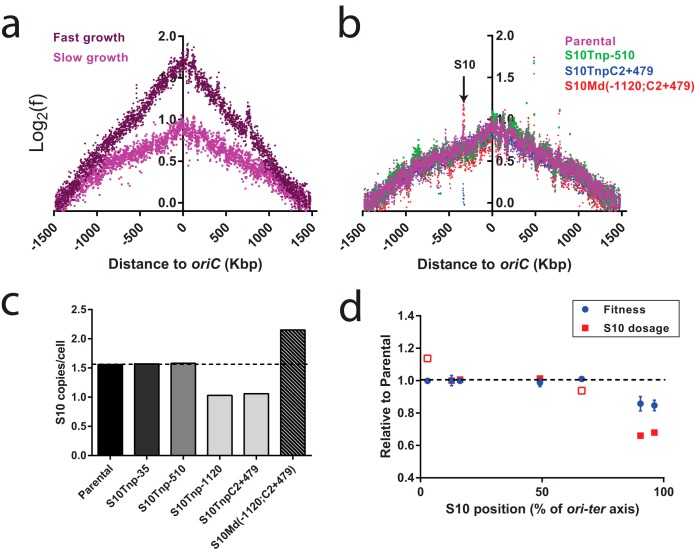
S10 relocation causes a dosage reduction in the absence of overlapping replication rounds that correlates with fitness impairment. MFA profiles are obtained by plotting the normalized number of reads at each position in the genome as a function of the relative position on the *V. cholerae* main chromosome with respect to *ori1* (to reflect the bidirectional DNA replication) using an average of 1,000-bp windows. Windows containing repeat sequences were removed. (a) MFA profiles comparing the parental strain under fast- and slow-growth conditions, showing the presence (*ori1* frequency, >2) and absence (*ori1* frequency, <2) of multifork replication, respectively. (b) MFA profiles comparing the parental, movant, and merodiploid strains under slow-growth conditions. The arrow indicates the S10 position in the abscissa, evidencing its dosage alterations. (c) The S10 dosage for each strain in the absence of multifork replication was quantified with high precision using MFA data. The S10 dosage is the result of averaging the frequency of panels corresponding to the S10 locus and dividing it by the frequency of the 50 kbp flanking the *ter1* zone. (d) The fitness and the S10 dosage of each movant in the absence of multifork replication were plotted as a function of the *ori*-*ter* axis of the *V. cholerae* genome. Blue circles correspond to the *W*_rel_ of each strain. Red squares correspond to the S10 dosage calculations from MFA experiments. Filled squares correspond to data directly obtained by MFA, while open squares mean that the data were inferred from the other data sets (see Materials and Methods).

The results above suggest that the fitness cost associated with S10 relocation far from *oriC* under slow-growth conditions is the result of a lower dosage of this essential locus. However, such small copy number differences are technically challenging to detect by conventional methods such as quantitative PCR (qPCR). Marker frequency analysis (MFA), which consists of deep-sequencing the genomic DNA in exponentially growing cells and then aligning and counting the reads against the genome of *V. cholerae* (see Materials and Methods) (34), enables the detection of slight dosage differences. The frequency of each locus across the *ori-ter* axis goes from a maximum at *ori1* to a minimum in the *ter* region across a solid line (21, 34). A discontinuity of this line evidences a dosage alteration or a deletion in that region of the genome (34). The average number of replication forks per cell is quantified by the *ori1/ter1* ratio. We first compared the MFA profiles of the parental strain under fast- and slow-growth conditions (Fig. 4a). As expected, the two profiles differed markedly. Under fast-growth conditions, we noticed an increased slope and *ori1* frequency as a consequence of the higher replication rate and increased overlapping replication rounds. Fast-growing bacteria harbored an average of 3.51 *ori1* copies per cell (Table S2), indicating that they were performing multifork replication. Slow-growing bacteria displayed an *ori1/ter1* ratio lower than 2 (~1.7 to 1.9), demonstrating the lack of overlapping replication rounds (Table S2). The comparison of the MFA profiles under slow-growth conditions revealed S10 dosage differences across different *V. cholerae* derivatives (Fig. 4b). The S10 locus displayed a dosage of ~1.5 copies per cell in the parental, S10Tnp−35, and S10Tnp−510 strains (Fig. 4c; Table S2) that is reduced to 1 copy per bacterium in S10Tnp−1120 and S10TnpC2+479 movants. In S10Md(−1120;C2+479), S10 dosage was increased to 2.15, beyond wild-type levels. In sum, in the absence of multifork replication, S10 dosage can vary from 1.5 to 1 copy per cell depending on its genomic position.

10.1128/mBio.00097-17.5 TABLE S2  Proportions between key genomic loci calculated from the MFA data. Download TABLE S2, PDF file, 0.1 MB. Copyright © 2017 Soler-Bistue et al. 2017 Soler-Bistue et al. This content is distributed under the terms of the Creative Commons Attribution 4.0 International license.

Next, we plotted *W*_rel_ values from pairwise competitions and the S10 dosage calculated from MFA as a function of S10 genomic position along the *ori-ter* axis (Fig. 4d). We observed that slight S10 dosage alteration either upward, as in S10Tnp+166, or downward, as in S10Tnp−510 and S10TnpC2+37, was well tolerated and did not impact fitness. Significant *W* loss was noticed only in S10Tnp−1120 and S10TnpC2+479 when S10 was very far from *ori1*, displaying its highest dosage reduction (~30%). Notably, a highly significant covariation between S10 dosage and strain fitness was detected using the two-tailed Pearson correlation coefficient (*r* = 0.91, *P* = 0.005). This supports the idea that, even in the absence of multifork replication, S10 genomic position can cause dosage differences that are big enough to impact cell fitness. Therefore, the S10 genomic location close to *oriC* results in a higher dosage that impacts bacterial fitness independently of the growth conditions.

## DISCUSSION

Bacterial chromosome organization is essential to compact a DNA molecule that is thousands of times larger than the cell. Meanwhile, the genetic material still needs to be replicated, transcribed, and segregated along the cell cycle. In this context, a body of evidence indicates that gene order within the bacterial chromosome contributes to genome organization and to coordinated cellular homeostasis with the cell cycle (11, 12, 14). Recent research describes several examples in which the genomic position of genes is essential to achieve their cellular functions (reviewed in reference 1). In most of them, phenotype alterations associated with gene relocation are the result of *oriC*-linked changes in genome-wide copy number (8, 10, 19, 35). In parallel, genes encoding transcriptional regulators alter many traits simultaneously, independently of their dosage (6). Other examples of genes whose expression is altered by dosage-independent effects are the ones regulated by H-NS (36), genes affected by HU (16), and those overlapping chromosome structural organization features (2).

RP, rRNA, some tRNAs, and RNAP genes are biased toward *oriC* in fast-growing bacteria (4, 9, 13). It has been proposed that the advantage of such location bias (Fig. 1a) is increasing their dosage during fast growth by recruiting multifork replication (4, 9). We recently showed that the interplay existing between S10 location, µ, and bacterial infectivity is mostly due to changes in its dosage (Fig. 1c) (19). However, several facts led us to hypothesize that an S10 location close to *oriC* may provide benefits other than a higher dose during fast growth. First, S10 harbors a large cluster of some of the most highly expressed genes (37). Its actual location impacts local supercoiling, contributing to the organization of the nucleoid structure (26, 27). On the other hand, the ribosome is an ~2.5-MDa complex consisting of 3 RNA molecules and some 50 different proteins that must be assembled in a precise order (38, 39). Therefore, S10 location could be important if their products are required in *cis* for ribosome biogenesis, in the context of a crowded cytoplasm (28, 30) in which mRNAs remain close to their encoding genes (7, 40). Toward this aim, we analyzed a set of isogenic S10 movants (Fig. 1b) using pairwise competition analyses. In this experimental approach, we expected to detect fitness differences not obvious by μ measurements, as the strains are striving to occupy the same niche, making differences displayed across lag, exponential, and stationary phases play a role. Slow-growth conditions should maximize the chances to uncover dosage-independent effects since (i) multifork replication is avoided, uncoupling S10 genomic position from its dosage, and (ii) a lower metabolism reduces the cytoplasm fluidity (32), slowing the diffusion of the ribosomal components. Interestingly, since *rpoA* and *secY*, the sole S10 genes that are not RP, are also part of macromolecular complexes (i.e., the transcription machinery and the translocon), they might also benefit from dosage-independent effects.

Under fast-growth conditions, results from pairwise competitions did not greatly differ from those determined by µ (Fig. 2a; Table 1). Moreover, growth curves seem to be more sensitive since strains S10TnpC2+37 and S10Md(−1120;C2+479) showed small μ reductions (4.8% and 3.07%, respectively) but no significant fitness reductions (*W* = 0.99 and *W* = 1.01, respectively). The fact that a slightly smaller μ during exponential phase could be compensated by a better survival during stationary phase or a shorter death phase could explain these differences. Reversion by suppressor mutations seems unlikely in such a short-term experiment. Although the two strains show similar S10 expression levels and a significant μ reduction, S10Tnp−510 and S10TnpC2+37 display different behaviors in competition experiments. The lack of *W* impairment in S10TnpC2+37 might be explained by suppressor mutations compensating S10 expression reduction. Alternatively, S10Tnp−510 generation coselected mutations contributing to a lower fitness. Since many of the strains [S10Tnp+166, S10Tnp−35, S10Tnp−510, S10TnpC2+37, S10Md(−510;−1120) and S10Md(−1120;C2+479)] showed no fitness impairment (Fig. 2) or μ reduction (Tables 1 and 2) (19), eventual alterations in chromosome structure due to S10 heterologous position do not constitute a burden for the cell. This also means that the precise position of the locus is not vital. This is in line with the observations done in *Mycoplasma mycoides* (JCV-Syn3.0) in which the exact position of essential genes was not relevant (41). Meanwhile, competition experiments under slow-growth conditions revealed that relocating S10 close to *ter* imposed a fitness handicap that could be completely rescued by the addition of a second *ori1-*distal copy (Fig. 2b). MFA confirmed that, in the absence of multifork replication (Fig. 4a), the S10 genome-wide copy number was altered by its relocation (Fig. 4b and c). S10 dosage changes across movant strains closely correlated with their fitness (Fig. 4d). This shows that, at least under laboratory conditions, dosage-independent effects are very unlikely. An alternative approach to uncover them would be using S10Tnp−1120 to relocate one or more rRNA operons along with the rest of the RP loci close to *ter1*. Although unlikely, a full or partial recovery on µ or on fitness could indicate that spatial effects also exist. The S10 genomic location provides the benefit of a higher dosage in the absence of multifork replication, suggesting that the selective pressure for RP positioning in *V. cholerae* has been exerted both in the amplification and in the persistence stages of this microorganism’s life cycle. This must be true for the members of *Vibrionaceae*, where the S10 position close to *ori1* is conserved (19). Our results imply that RP positioning might also influence the evolution of slow-growing bacteria.

We also explored if S10 dosage could be further increased. If the genomic position of the locus is already optimized, increasing its dosage should be detrimental for cell physiology. Alternatively, if its copy number is limiting metabolism, an S10 dosage increment might improve bacterial growth. Even though up to four S10 copies were tolerated, *V. cholerae* physiology could not be boosted (Fig. 3). This is in line with the notion that the rate-limiting step in ribosome biosynthesis is rRNA abundance, which in turn regulates RP expression (42). An S10 copy number beyond 2 was detrimental for the cell (Fig. 3). Such physiological impairment may be the consequence of an imbalance in the cellular composition of ribosomal proteins, making the RP outside the S10 locus limiting in ribosome biogenesis (43). Also, the excess in S10 dosage might overcome the many inhibition mechanisms (38, 42, 44), leading to a futile and energetically costly overexpression. These results support the idea of an evolutionarily optimized S10 dosage. In this line, a recent study shows that the addition of extra rRNA operons cannot increase the growth rate in *Escherichia coli* (45). Similarly, addition of rare tRNAs is detrimental for *E. coli* growth (46). Meanwhile, *V. cholerae* seems to cope better with excess than with lack of S10, since merodiploid strains showed no impairment (Fig. 3) while a mere 30% of dosage reduction impacted cell fitness. This is in line with the fact that RP are regulated by the availability of rRNA, via a translational feedback mechanism (42, 44, 47, 48), which is able to buffer the dosage excess across a relatively wide range. A dosage reduction cannot be counterbalanced.

Overall, our observations rule out, or at least downplay, the existence of dosage-independent effects for the S10 locus. We believe that its extremely high expression (37) neutralizes the “spatial address effect” (3, 49, 50) by generating a high bioavailability of S10 proteins across the whole cellular space. Meanwhile, the high requirement for S10-encoded products makes even relatively small dosage reductions impact cell fitness. We believe that other genes encoding the pathways for the expression of genetic information, such as other RP loci, rRNA, tRNA, and RNAP, should face a similar scenario.

This work deepens our understanding of how the location of strategic genes can influence the evolution of bacteria. There is an increasing number of genes whose genomic position impacts the encoded phenotype (1, 6, 8, 10, 15, 19, 36) that seem to be the tip of the iceberg of many more to discover. As a general rule, some genes change their dosage according to the genomic location due to *oriC*-linked changes in their genome-wide copy number (1). On the other hand, the positioning of some genes could provide an adequate subcellular location (6, 36, 40). 

More studies employing positional genetics approaches need to be done to better understand this issue. Such studies will help in discovering the rules of genome organization which are essential in the context of the creation of the first artificial life forms (41) that will permit rational design of the genomes of microorganisms with specific properties (51).

## MATERIALS AND METHODS

### Bacterial strains, plasmids, and culture conditions.

For fast-growth conditions, bacterial cultures were done in Lennox Luria broth (LB) at 37°C with agitation at 200 rpm. Slow growth was performed on M9 minimal medium at 30°C supplemented with 0.4% glucose as carbon source, 0.1 mM CaCl_2_, and 1 mM MgSO_4_, with agitation at 200 rpm. For selection, the following antibiotics were used: chloramphenicol (3 μg/ml), kanamycin (25 μg/ml), spectinomycin (100 μg/ml), carbenicillin (50 μg/ml), and zeocin (25 μg/ml). Strains and plasmids used in this study are listed in Table S1 in the supplemental material. Details on meropolyploid generation are in Text S1 in the supplemental material.

10.1128/mBio.00097-17.1 TEXT S1 Supplemental methods. Download TEXT S1, PDF file, 0.2 MB. Copyright © 2017 Soler-Bistue et al. 2017 Soler-Bistue et al. This content is distributed under the terms of the Creative Commons Attribution 4.0 International license.

### General procedures.

Genomic DNA was extracted using the GeneJET genomic DNA purification kit, while plasmid DNA was extracted using the GeneJET plasmid miniprep kit (Thermo Scientific). PCR assays were performed using Phusion high-fidelity PCR master mix (Thermo Scientific).

### Automated growth curve measurements.

Overnight (ON) cultures of the indicated microorganism were diluted 1/1,000 in LB and incubated at 37°C. Bacterial preparations were distributed by triplicate or quadruplicate in p96 microplates. Growth-curve experiments were performed using a Tecan Inﬁnite Sunrise microplate reader, with absorbance measurements (600 nm) taken at 5-min intervals for 12 h on agitation. Slopes during exponential phase were directly obtained using a home-made Python script coupled to the GrowthRates program (52).

### Competitive fitness assays.

The assays were done as described previously (53). Briefly, the fitness of each strain was measured relative to the Parental−1120::gfpmut3* strain (54) (see Table S1). Each strain was cultured overnight at 37°C with 200-rpm shaking in 3 ml of LB broth. After being diluted 200-fold, 5 µl was measured by flow cytometry (FC) in a MACSQuant Analyzer 10 (Miltenyi Biotec, Inc.) to assess the amount of cells and to verify the fluorescence of green fluorescent protein (GFP)-expressing *V. cholerae*. Then, all cultures were mixed at a ratio of 1:1 with the latter strain. The initial proportions were confirmed by FC, and mixtures were diluted ~10^5^-fold in fresh LB or 10^3^-fold for M9 and competed for 18 h at 37°C or 30°C, respectively, with shaking at 200 rpm (≈26 generations and 12 generations, respectively). The final proportion was obtained by FC. The fitness of each mutant relative to the GFP-expressing *V. cholerae* strain was determined using the formula *W*_mutant_ = ln(*N*_final_/*N*_initial_)/ln(*N*_gfp_._final_/*N*_gfp_._initial_), where *W*_mutant_ is the fitness of the derivative strain under study, *N*_initial_ and *N*_final_ are the quantity of the derivative strain before and after the competition (nonfluorescent), respectively, and *N*_gfp.initial_ and *N*_gfp.final_ are the numbers of cells of Parental−1120::gfpmut3* before and after the competition, respectively. The experiments were performed at least 3 times using 5 or more biological replicates. Relative fitness (*W*_rel_) is the ratio of the *W* of each derivative and the *W* of the parental strain.

### Genome engineering.

To build meropolyploid strains, we employed previously developed genome engineering tools (19, 55) and the natural transformation capacity of *V. cholerae* (56–58). More details can be found in Text S1 in the supplemental material. Briefly, using plasmid pCP20 as described before (19, 55), we removed antibiotic resistance markers flanking S10 from Parental+166 to obtain Parental+166Δ(*aph*,*cat*). Then, we built S10Tnp+166Δ(*aph*,*cat*) by S10 relocation using plasmid pMP96. This strain was then successively transformed with genomic DNA from PGB-B393, S10Tnp−35Δ*aph*, and S10TnpC2+37Δ*cat* (see Table S1 and Text S1). Transformants were selected in zeocin, chloramphenicol, and kanamycin. Genotype was checked by Southern blotting (Fig. S1 and Text S1). Strains were built several times independently, and we found no evidence that observed phenotypes resulted from the accumulation of suppressor mutations.

### MFA.

Genomic DNA extracted from early exponential phase (optical density at 450 nm [OD_450_] of ~0.15) was used for library preparation using a PCR-free protocol. Libraries were sequenced on an Illumina MiSeq sequencer using 100- to 150-base-length paired-end reads for 100× genome coverage. The resulting FastQ files were analyzed using R2R script to obtain the frequency of each locus along the genome (21, 34). The log_2_ frequencies every 1,000-bp window were then plotted as a function of replichore length. After marker frequency analysis (MFA), *ori1* and *ter1* were quantified by averaging 50 frequency data points corresponding to *ori1* and *ter1* zones. The *ori2* and *ter2* frequencies were obtained by averaging 20 data points, due to the smaller size of the secondary chromosome. The S10 frequency was calculated by averaging panels corresponding to VC2569 and VC2599, respectively. These values were used to calculate S10 dosage with high precision by calculating the S10/*ter1* ratio. For strains S10Tnp+166 and S10TnpC2+37, the S10 frequency was estimated by using the frequency of the 10 kbp flanking the insertion site of the locus (intergenic space between VC2739 and VC2740 and between VCA0030 and VCA0031, respectively) in the parental strain and in the S10TnpC2+479 movant data sets.
