# Supplementary material for: The Proximity of Ribosomal Protein Genes to oriC Enhances Vibrio cholerae Fitness in the Absence of Multifork Replication
Source: mBio. 2017 Feb 28;8(1):e00097-17. doi: 10.1128/mBio.00097-17 (PMC5347342; doi:10.1128/mBio.00097-17)
Supplement: TABLE S1 [file mbo001173213st1.pdf]

Table S1. Full list of plasmids, bacteria strains used:

Name	Relevant genotype or features	Reference
**Plasmids**		
pMP96	pSC101*rep*^{TS} *oriT*$_{RP4}$ [int_{λ}-xis_{λ}, int_{HK}-xis_{HK}]	Val et al. 2012
pCP20	pSC101*rep*^{TS} [*flp*]	Cherepanov et al. 1995
pASB11	pCR-BluntII-Topo:: lox66-Zeo-lox71	Soler-Bistué et al. 2015
pJBA28	delivery plasmid for mini-Tn5-Km-PA1/04/03-RBSII-*gfpmut3**-T0-T1, Amp^{R} and Kan^{R}	Andersen JB et al 1998
pASB21	pCR-BluntII-Topo:: (gfpmut3**Not*I fragment-lox66-Zeo^{R}-lox71)	This study
Escherichia coli		
DH5α	F^{-} *endA1 glnV44 thi-1 recA1 relA1 gyrA96 deoR nupG* Φ80d*lacZ*ΔM15 Δ(*lacZYA-argF*)U169, hsdR17(r_K^- m_K^+), λ^-	
Vibrio cholerae		
N16961*ChapRΔlacZ*	N16961::mTn*7hapR*$^{+}$ Δ*lacZ* using pMEV69	Val et al. 2012
Parental -1120	PGB-A192::*attB*'-*lox66-dfrB1-lox71* inserted in the intergenic region between VC1508-VC1509.	Soler-Bistué et al. 2015
Parental C2+479	PGB-A192::*attB*'-*lox66-dfrB1-lox71* inserted in the intergenic region between VCA0543-VCA0544.	Soler-Bistué et al. 2015
PGB-B393	N16961 Δ*lacZ,* [lox2272-*ere(A)*-lox2272] (at the intergenic space VC2569-VC2570), [*lox66-zeo*^{R}*-lox71*] (at the intergenic space VC2599-VC2600) flanking the region VC2569 and VC2599 encompassing *S10-spc-α* locus.	Soler-Bistué et al. 2015
S10Tnp+166	S10 relocated closer to *oriC1* in the intergenic region between VC2739-VC2740.	Soler-Bistué et al. 2015
S10Tnp-35	S10 relocated next to its original location in the intergenic region between VC2536-VC2537.	Soler-Bistué et al. 2015

S10Tnp-510	S10 relocated at the middle of the left replichore of chromosome 1 in the intergenic region between VC2075-VC2076.	Soler-Bistué et al. 2015
S10Tnp-1120	S10 relocated near the *dif* region of chromosome 1 in the intergenic region VC1508-VC1509.	Soler-Bistué et al. 2015
S10TnpC2+37	S10 relocated near the *oriC2* in the intergenic region between VCA0030-VCA0031.	Soler-Bistué et al. 2015
S10TnpC2+479	S10 relocated near the *dif* sequence of chromosome 2 in the intergenic region between VCA0543-VCA0544.	Soler-Bistué et al. 2015
PGB-B393	N16961 Δ*lacZ*, [lox2272-*ere(A)*-lox2272] (at the intergenic space VC2569-VC2570), [*lox66-zeo*^{R}*-lox71*] (at the intergenic space VC2599-VC2600) flanking the region VC2569 and VC2599 encompassing *S10-spc-α* locus.	Soler-Bistué et al. 2015
S10Md(-510;-1120)	Merodiploid bearing *S10-spc-α* copies at the intergenic sequences of VC2075-VC2076 and VC1508-VC1509.	Soler-Bistué et al. 2015
S10Md(-1120;C2+479)	Merodiploid bearing *S10-spc-α* copies at the intergenic sequences of VC1508-VC1509 and VCA0543-VCA0544.	Soler-Bistué et al. 2015
Parental +166 Δ(*aph,cat*)	PGB-A192::*attB*'*-lox66-dfrB1-lox71* Δ(*aph,cat*) inserted in the intergenic region between VC2739-VC2740. Kanamycin and chloramphenicol resistance cassettes were deleted using a flipase expressing plasmid.	This study
S10Tnp+166 Δ(*aph,cat*)	S10 relocated closer to *oriC1*. Derived from Parental+166 Δ(*aph,cat*).	This study
S10Tnp-35 Δ(*aph*)	S10Tnp-35 kanamycin resistance cassettes were deleted using a flipase expressing plasmid.	This study
S10TnpC2+37 Δ(*cat*)	S10TnpC2+37 chloramphenicol resistance cassettes were deleted using a flipase expressing plasmid.	This study
S10M2(+166;0)	Merodiploid bearing 2 *S10-spc-α* copies at the intergenic regions between of VC2739-VC2740 and at its original location.	This study
S10M2(+166;-35)	Merodiploid bearing 2 *S10-spc-α* copies at the intergenic regions between of VC2739-VC2740 and VC2536-VC2537.	This study

S10M2(+166;C2+37)	Merodiploid bearing 2 *S10-spc-α* copies at the intergenic regions between of VC2739-VC2740 and VCA0030-VCA0031.	This study
S10M3(+166;0;C2+37)	Merotriploid bearing 3 *S10-spc-α* copies at wild type location plus two copies at the intergenic sequences between ORFs VC2739-VC2740 and VCA0030-VCA0031.	This study
S10M3(+166;0;-35)	Merotriploid bearing 3 *S10-spc-α* copies at wild type location plus two copies at the intergenic sequences between ORFs VC2739-VC2740 and VC2536-VC2537.	This study
S10M4(+166;0;-35;C2+37)	Merotetraploid bearing 4 *S10-spc-α* copies, one at wild type location and 3 others at the intergenic sequences between VC2739-VC2740, VC2536-VC2537 and VCA0030-VCA0031.	This study
V.cholerae::*gfpmut3**	NotI fragment from pJBA28 (Andersen et al. (1998)) containing promoter $P_{A1/04/3}$, RBSII and *gfpmut3** linked to Zeo^{R} from pASB21 gene was inserted in the intergenic region between VC0696-VC0697.	This study
Parental-1120::*gfpmut3**	*gfpmut3**-ZeoR cassette was inserted in the intergenic region between VC0696-VC0697 in Parental-1120 strain.	This study
