# Supplementary material for: The Proximity of Ribosomal Protein Genes to oriC Enhances Vibrio cholerae Fitness in the Absence of Multifork Replication
Source: mBio. 2017 Feb 28;8(1):e00097-17. doi: 10.1128/mBio.00097-17 (PMC5347342; doi:10.1128/mBio.00097-17)

Figure S1: Southern Blot of *Eco*RV digested gDNA of the strains indicated in the upper panel. Probes were targeted to the *rpsJ* gene (red, DY682) or to the Zeo^R marker-linked to the parental *rpsJ* gene from PGB-B393 strain (green, DY782). Genotype changes are evidenced by size change of S10 upon movement (parental vs S10Tnp+166). Then the addition of the second and third copy from S10Tnp-35Δ*aph* and S10TnpC2+37Δ*cat* (Black arrows). The fourth S10 copy comes from PGB-B393. The restriction fragment has a similar size than the +166 allele. However, it can be distinguished by an increased S10 probe signal (red arrow) and the green band. S10 Ploidy of each strain is showed in the lower panel.

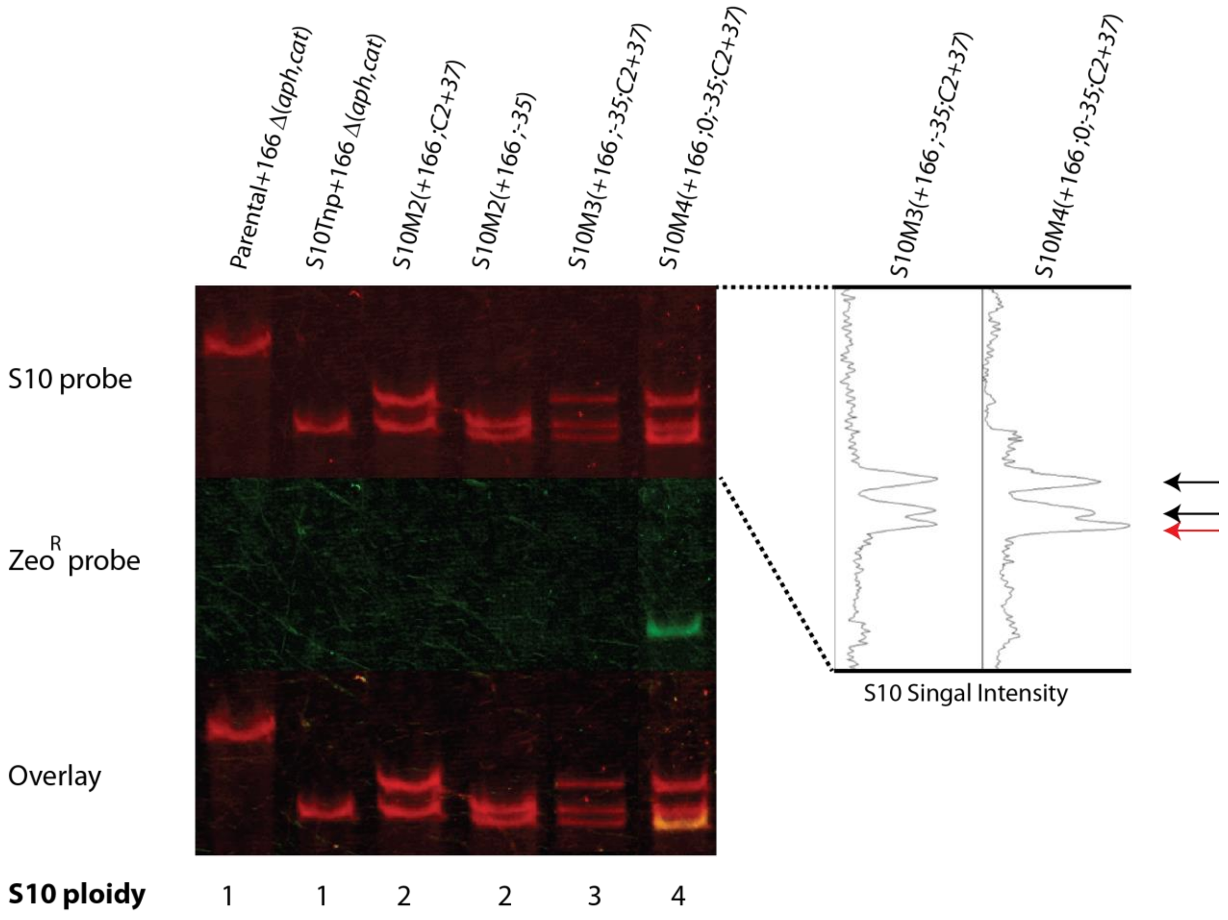

Supplement: FIG S1 [file mbo001173213sf1.pdf]
