# Supplementary material for: The Proximity of Ribosomal Protein Genes to oriC Enhances Vibrio cholerae Fitness in the Absence of Multifork Replication
Source: mBio. 2017 Feb 28;8(1):e00097-17. doi: 10.1128/mBio.00097-17 (PMC5347342; doi:10.1128/mBio.00097-17)

Figure S2: Analysis of growth rate of meropolyploids. The S10 ploidy effect on GR was quantified by averaging obtained µ in at least 2 independent experiments, with 4 or more biological replicates, for each mutant strain and normalizing it to the µ of the parental strain. Results are expressed as percentage of the variation (µ %) with 95% CI with respect to parental strains. Statistical significance was analyzed by one-way ANOVA two-tailed test. Then Dunn test was made for multiple comparisons taking the Parental strains as control. Statistically significant differences are indicated as, *** meaning p< 0.001 and**** which stands for p<0.0001.

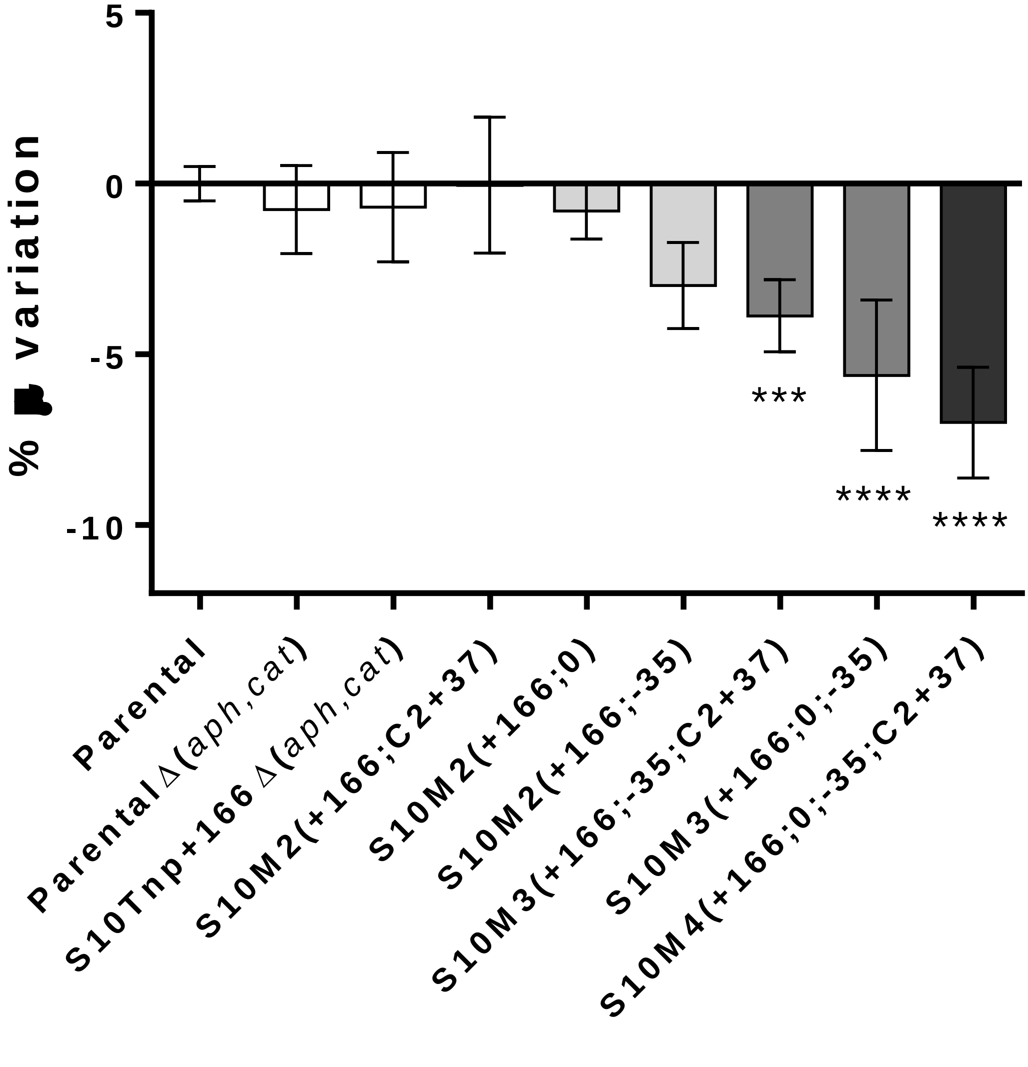

Supplement: FIG S2 [file mbo001173213sf2.pdf]
