# Supplementary material for: The Proximity of Ribosomal Protein Genes to oriC Enhances Vibrio cholerae Fitness in the Absence of Multifork Replication
Source: mBio. 2017 Feb 28;8(1):e00097-17. doi: 10.1128/mBio.00097-17 (PMC5347342; doi:10.1128/mBio.00097-17)
Supplement: TABLE S2 [file mbo001173213st2.pdf]

Table S2: Proportions between key genomic loci calculated from the MFA data.

	Parental Fast growing conditions	Slow growing conditions					
		**Parental**	**-35**	**-510**	**-1120**	**C2+479**	**(-1120;C2+479)**
ori1/ter1	3.51	1.74	1.79	1.94	1.73	1.81	1.84
ori2/ter2	1.69	1.27	1.29	1.28	1.31	1.55	1.31
ori1/ori2	2.16	1.33	1.35	1.49	1.33	1.32	1.47
S10/ter1	2.63	1.56	1.57	1.58	1.03	1.06	2.15
ori1/S10	1.33	1.12	1.14	1.23	1.68	1.71	0.86
