# Supplementary material for: The Proximity of Ribosomal Protein Genes to oriC Enhances Vibrio cholerae Fitness in the Absence of Multifork Replication
Source: mBio. 2017 Feb 28;8(1):e00097-17. doi: 10.1128/mBio.00097-17 (PMC5347342; doi:10.1128/mBio.00097-17)
Supplement: TEXT S1 [file mbo001173213s1.pdf]

Appendix: Supplementary Methods

S10 meropolyploid generation. Parental+166 strain was transformed with pCP20 plasmid and selected on LB agar plates supplemented with carbenicillin. Transformed clones were cultured ON at 30°C for *aph* and *cat* removal by recombination reaction catalyzed by Flp. Then, individual clones were selected from LB-agar plates and restreaked on plates supplemented with kanamycin, with chloramphenicol and LB. Clones showing a Kan^S Cm^S phenotype were then grown ON in liquid LB at 37°C to promote pCP20 plasmid loss. Culture was restreaked on LB-agar plates to obtain isolated clones susceptible to carbenicillin. Thus, we obtained Parental +166 Δ(*aph,cat*) strain. S10 transposition to the intergenic region between VC2739-VC2740 was done described before (Soler-Bistué et al 2015) to get S10Tnp+166 Δ(*aph,cat*). This movant was induced for natural compentence and transformed with gDNA from PGB-B393. Zeo^R clones were selected on LB-agar plates supplemented with the adequate antibiotics to produce S10M2(+166;0). S10 merotriploid strains were obtained by naturally transforming this latter derivative with genomic DNA from , S10Tnp-35 Δ(*aph*) or S10TnpC2+37 Δ(*cat*). These latter strains were obtained by transforming , S10Tnp-35 and S10TnpC2+37 with pCP20 plasmid, culturing the transformant strains for 15 minutes at 30°C for either *aph* or *cat* removal by Flp recombination. S10 merotetrapolyploids, S10M4(+166;0;-35;C2+37), were obtained by transforming the S10 merotriploids with and additional copy of S10. For this S10M3(+166;0;-35) was transformed with gDNA from S10TnpC2+37 Δ(*cat*) then selected in LB Kanamycin and S10M3(+166;0;-35) was transformed with gDNA from S10Tnp-35 Δ(*aph*) and selected in LB supplemented with chloramphenicol. Antibiotic resistance profile was checked at each step to avoid the loss of S10 copies inserted in the previous steps. The final genotype was further confirmed by Southern Blot. The strains were built and checked many times independently.

Southern Blot. DNA was extracted from 0.4 mL of an ON bacterial culture. DNA was dried on a speedvac device and then was digested with *Eco*RV. Samples were resolved by gel electrophoresis in 1X TAE buffer. After drying the gel for 1h at 60°C, DNA was denatured by rinsing in Denaturation Solution, NaOH 0.5 M, NaCl 0.15 M for 20 minutes at room temperature. The gel was equilibrated using Neutralization Solution, Tris 0.5 M pH=8 NaCl 0.15 M for 20 min. Then pre-hybridization was performed for 30 minutes at 50°C with 10 mL of RapidHyb Buffer (GE). Next, hybridization was performed ON at 50°C with 1,5 uL of 10mM of a 40 nucleotide long DNA probe fluorescently labeled with DY682 or DY782 dyes. Finally, washes were performed once in 6X SSC, 0.5% SDS at 50°C, and twice for 20 minutes at 55°C. A final wash was performed for 15 minutes at 60°C. Then gel was read using an Odyssey infrared imaging system was used. Analysis of band density was done using FIJI (formerly known as ImageJ).

<u>Probes used</u>:

S10hybr5’: targeting *rpsJ* gene.

5’-TCACGACTCGGAATTGGATTGTCATAATTGTCGGCTTCGC-3’-DY682

ZeoDY782_X2: targeting zeo^R gene that is linked to *rpsJ*.

5'DY782-CAAAATCATCTTCCACAAAATCGCGGCTAAAACCCAGACG-3’ DY782

Cat-DY682_X2: targeting *cat* gene that is linked to *rpsJ*.

5'DY682-GTATTCATTAAGCATCTGCCGACATGGAAGCCATCACAAA-3’ DY682
